# Huge Intra-abdominal Retroperitoneal Spindle Cell Mass in a Young Adult: A Report of a Rare Case and Literature Review

**DOI:** 10.7759/cureus.67822

**Published:** 2024-08-26

**Authors:** Fathi Elgeyoushy, Amal Sayfuldeen Qari, Mohammed A Al-Refai, Tarek Hamed Taweek, Hatem Hussain Alharbi

**Affiliations:** 1 General Surgery, King Fahad Hospital, Medina, SAU; 2 Medicine, King Fahad Hospital, Medina, SAU; 3 General and Thoracic Surgery, King Fahad Hospital, Medina, SAU

**Keywords:** spindle cell neoplasm, oncology, intrabdominal, huge, abdominal mass

## Abstract

GI stromal tumors (GISTs) are the most common mesenchymal neoplasms in the GI tract. GISTs are malignancies that typically originate in the digestive system, most often in the stomach and small intestine. Histopathological classification identifies three types of GISTs: spindle cell, epithelioid, and mixed. We present a case of a huge intra-abdominal retroperitoneal mass in a 23-year-old female with no notable medical or surgical history. She experienced dysphagia and early satiety for one year but did not seek medical attention until presenting at our clinic. Her abdomen was distended, soft, and firm, with a huge non-tender mass. Abdominal CT revealed a substantial left retroperitoneal soft tissue lesion measuring 17 × 12 × 21 cm, causing a significant mass effect. An exploratory laparotomy via a thoracoabdominal approach was performed. A definitive diagnosis required surgical intervention or tissue biopsy. This case highlights the rarity of spindle cell neoplasms and underscores the importance of maintaining a high index of suspicion for such tumors in young adults.

## Introduction

GI stromal tumors (GISTs) are the most prevalent type of mesenchymal neoplasm found in the GI tract. The World Health Organization categorizes tumors of probable origin into over 100 histological types based on the architectural pattern of soft tissue neoplasms [1]. GI mesenchymal tumors include various spindle cell cancers, such as fibromatoses, inflammatory fibroid polyps, schwannomas, leiomyomas, and GISTs. Among these, GISTs are the most significant subset of mesenchymal tumors in the GI tract. GIST cells are typically spindle-shaped (70%), but some GISTs consist of epithelioid cells (20%) or a mixture of cell types [2]. Mazur and Clark first described GISTs as mesenchymal gut tumors originating from the myenteric nerve system in 1983. In the United States, 3,300 to 6,000 new cases are reported annually, while Saudi Arabia has seen an increase in incidence from 2.1 per million in 1995 to 12.7 per million in 2003 [3,4]. This case report presents a 23-year-old female patient with a giant retroperitoneal intra-abdominal mass, ultimately diagnosed as a spindle cell neoplasm.

## Case presentation

A 23-year-old female, with no significant medical or surgical history, presented to our general surgery outpatient clinic for the first time due to dysphagia and early satiety lasting for one year. She reported no weight changes, fever, night sweats, or bleeding from other orifices. There was no family history of congenital abnormalities. The patient had not sought medical attention until her visit to our clinic.

On clinical examination, no lymphadenopathy, jaundice, pallor, or pedal edema was observed, and all vital signs were within normal ranges. A bulging was noted in the left upper abdomen. Palpation revealed a huge, firm, non-tender, non-pulsatile swelling, with no redness or warmth. The mass extended from the left hypochondrium to the lumbar and umbilical regions, reaching dimensions that prevented us from inserting our hand between the mass and the costal margin.

Laboratory tests, including a complete blood count, urea and electrolyte levels, CRP, and liver function tests, were all within normal ranges.

**Figure 1:**
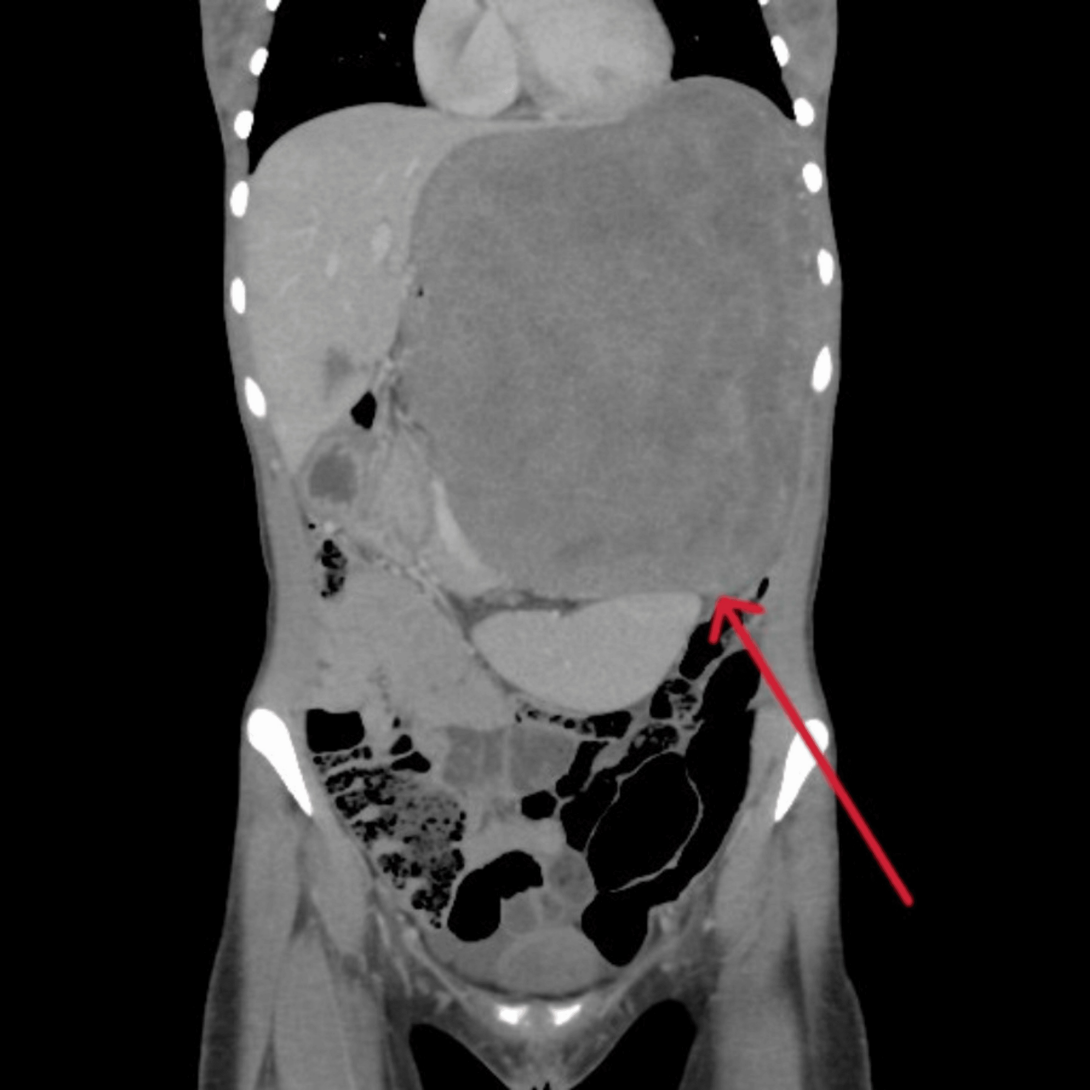
Coronal section of the abdomen and pelvis on CT The coronal cross-sectional CT image of this patient reveals a massive left retroperitoneal soft tissue lesion measuring 17 × 12 × 21 cm.

Following the history and physical examination, we ordered an abdominal CT scan (Figure 1). We informed the patient of the concerning results. The CT scan of the abdomen and pelvis with contrast revealed a huge left retroperitoneal soft tissue lesion, measuring 17 × 12 × 21 cm, causing a significant mass effect on adjacent organs. The mass was inseparable from the stomach and had herniated into the left thoracic cavity through a diaphragmatic defect. The displaced spleen exhibited homogeneous enhancement without focal lesions. No lymphadenopathy was observed (Figure 1).

The patient underwent an upper GI endoscopy prior to an exploratory laparotomy, which revealed normal mucosa in the esophagus and stomach with no evidence of infiltration. The pylorus appeared regular and reactive. An abdominal ultrasound (US)-guided biopsy was performed, which identified a hypocellular lesion composed of bundles and fascicles of bland spindle cells within an abundant fibromyxoid stroma. There was no significant atypia, mitosis, or necrosis observed. Immunohistochemical staining was positive only for smooth muscle actin; other stains were negative, leading to a final perioperative diagnosis of a spindle cell neoplasm.

**Figure 2:**
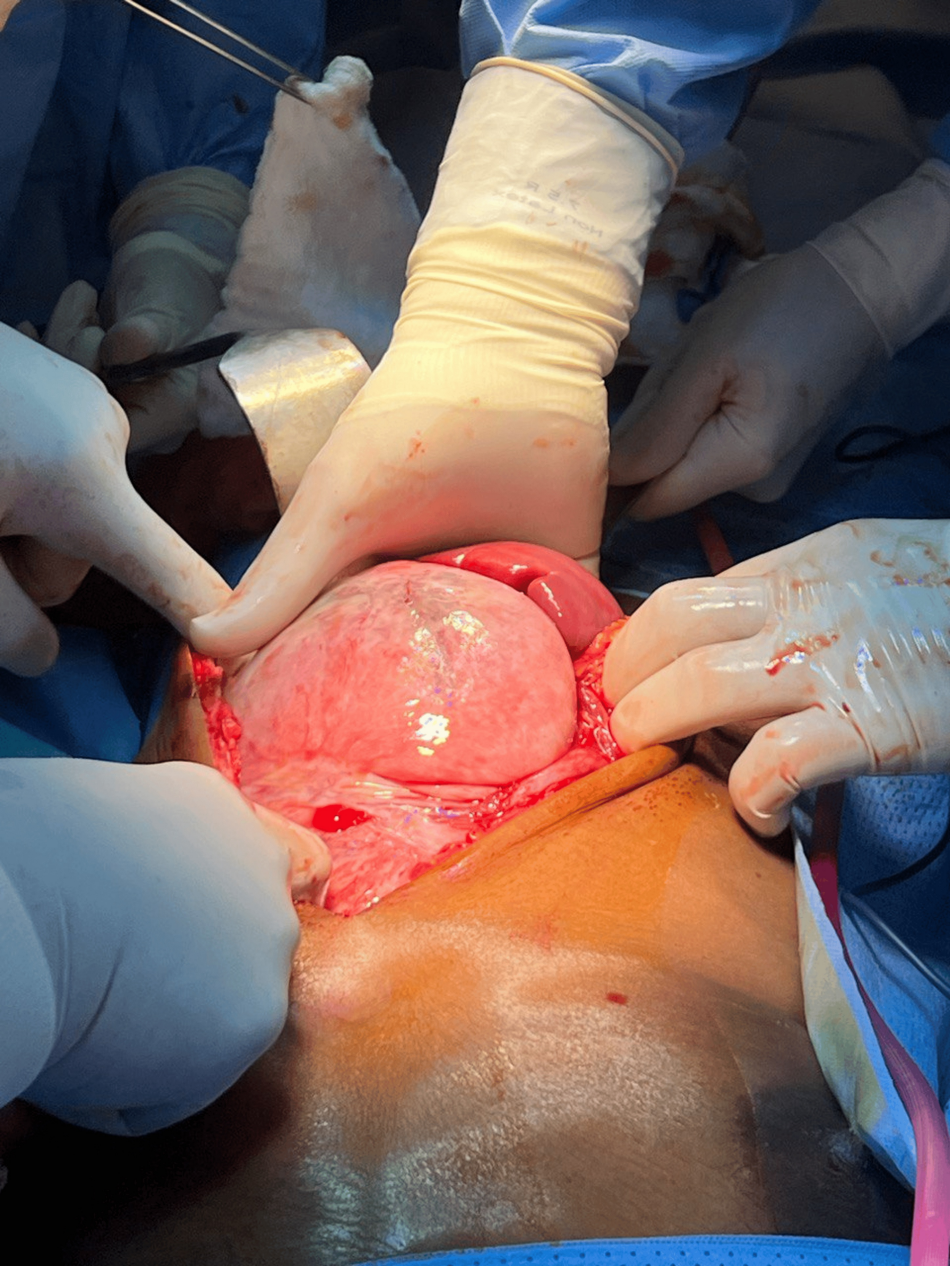
Exploratory laparotomy reveals a huge intra-abdominal retroperitoneal mass

**Figure 3:**
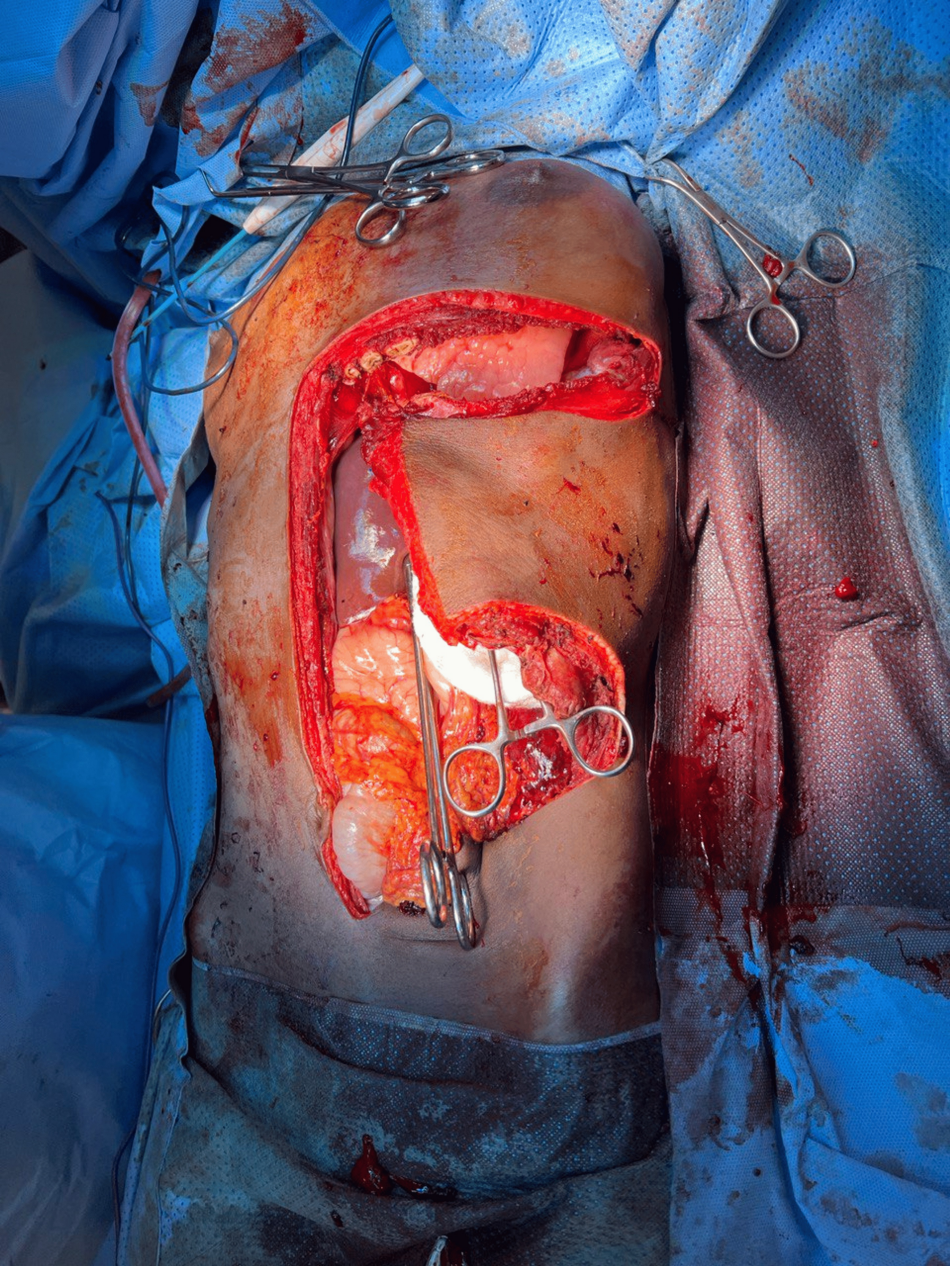
Exploratory laparotomy with a thoracoabdominal approach, featuring a T-shaped incision

**Figure 4:**
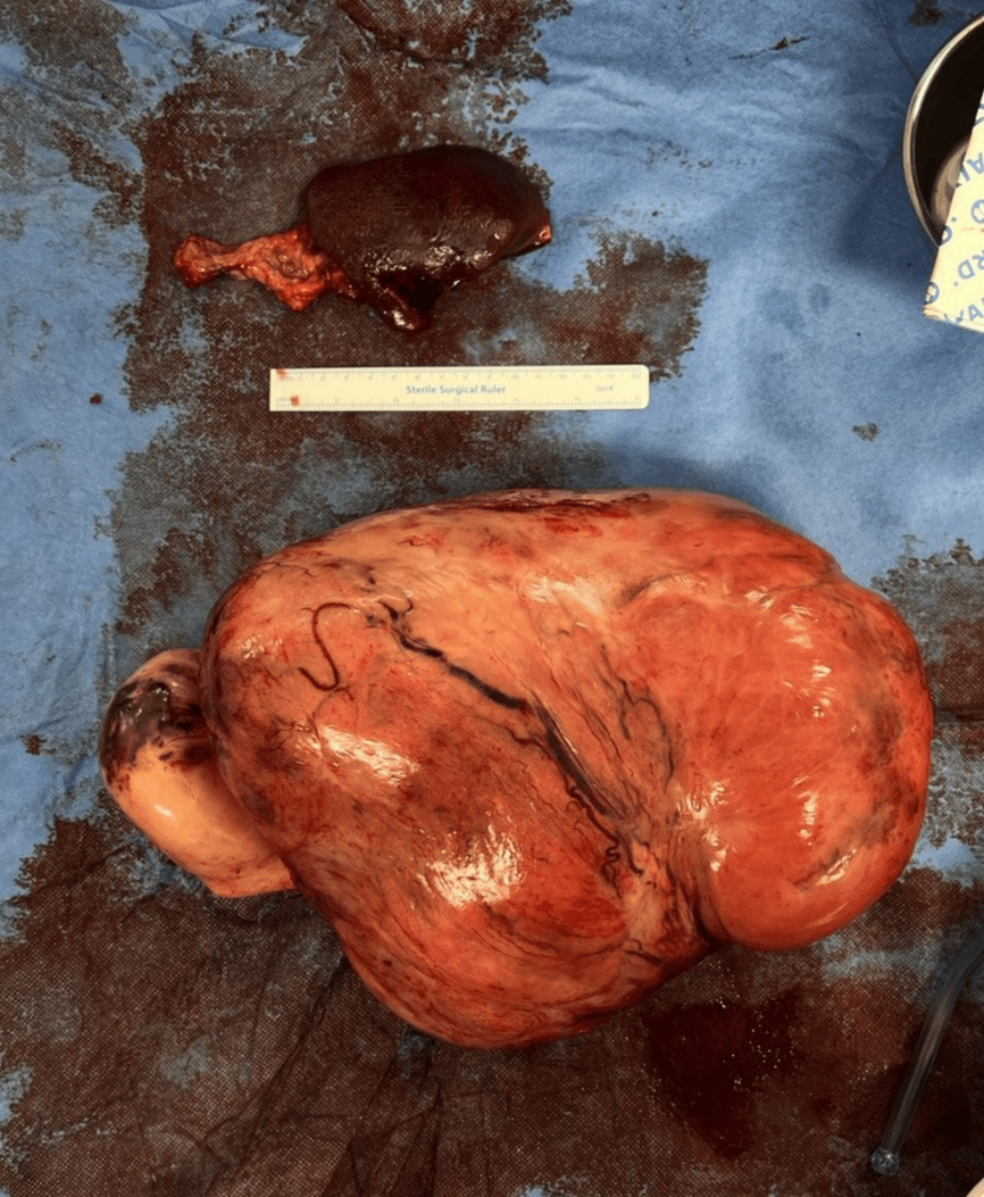
Surgical specimen Gross surgical specimen of the massive intra-abdominal retroperitoneal mass and spleen. The mass measures 17 × 12 × 21 cm.

Following the abdominal US-guided biopsy, the patient underwent exploratory laparotomy via a thoracoabdominal approach due to the huge retroperitoneal soft tissue lesion. A midline laparotomy incision was extended into a T-shaped incision with a left transverse component to accommodate the mass extending into the chest cavity. The findings included a large, smooth, highly vascular soft tissue mass occupying the left upper quadrant and extending to the umbilicus, displacing the spleen inferiorly toward the right. A splenectomy was performed because the spleen was displaced and inseparable from the mass (Figure 2, Figure 3, Figure 4).

The mass was attached to the gastroesophageal junction, and an injury occurred during dissection. After the mass was removed, the injury was repaired with a fundal patch, including primary suturing and application of the patch. The procedure concluded with the closure of the thoracotomy and the insertion of an intercostal tube.

**Figure 5:**
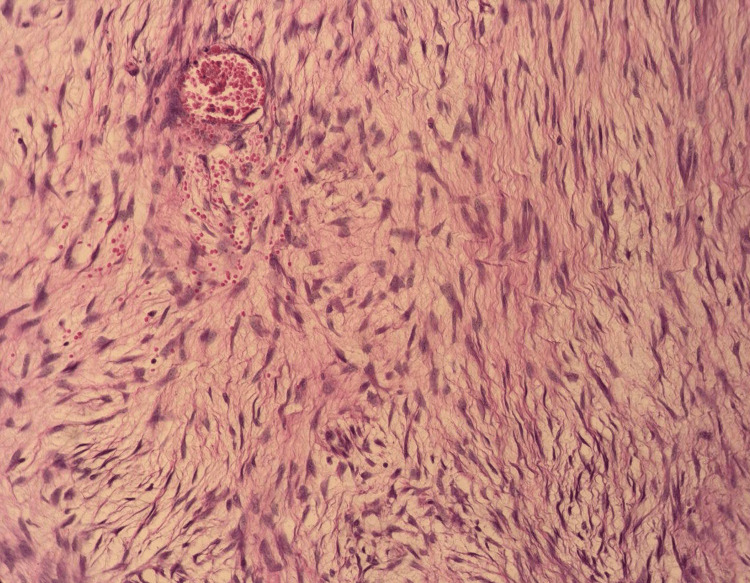
Magnified view of the stained pathological sections The specimen section is stained with H&E.

The patient was transferred to the ICU for close monitoring, and the mass specimen was sent for histopathological examination. The histopathology report revealed a benign spindle cell neoplasm characterized by bland cellular morphology and scattered small lumen vessels. The tumor exhibited areas of whirling and fasciculations with a myxoid background. There was no nuclear atypia, mitosis, epithelial component, necrosis, or cellular atypia observed (Figure 5).

The patient progressed well postoperatively and was discharged in stable condition after one week. She was referred for follow-up with oncology.

## Discussion

The stomach is the most common site for GISTs, which account for just 0.1-3% of all GI tumors [5]. Other frequent sites include the small intestine, colon, and rectum. GISTs can exhibit various histological patterns, including spindle cell, pleomorphic, and mixed types. The spindle cells in these tumors may be bland, regular, or markedly pleomorphic, and the architectural patterns can vary widely, including fascicular, storiform, lacelike, or myxoid. Occasionally, differentiation toward sarcomatous types such as osteosarcomatous or chondrosarcomatous can be observed. Unlike true sarcomas, spindle cell GISTs often show random, irregular fascicle development, and “transition-type” cells with mixed morphology may be present [6]. Recent studies indicate that 85-92% of GISTs have c-kit mutations [7].

A study of 244 soft tissue sarcoma patients at King Faisal Specialist Hospital & Research Centre in Riyadh revealed that males comprised 56.1% of the cohort, with a median age of 38 years. The most common histological subtypes were synovial sarcoma (19.6%), liposarcoma (13.5%), undifferentiated pleomorphic sarcoma (11.6%), and leiomyosarcoma (9.8%). Most patients (49.1%) presented with painless swelling, and the median duration of symptoms was 8 months (IQR: 4-13 months). Lung metastases were the most frequent, observed in 38.8% of cases [8]. Spindle cell sarcomas can affect individuals of any age or gender, with a median presentation age of 57 years reported by Feng et al. and Smith et al. Our patient was younger, presenting at 23 years old. The median tumor size reported by Smith et al. was 9.87 cm [9].

Common symptoms of GISTs include abdominal mass, GI bleeding, anorexia, and intestinal obstruction, although they are often discovered incidentally. In some cases, they may cause nonspecific symptoms like bloating and early satiety [10,11]. Diagnostic modalities such as CT, endoscopy, and double-contrast GI series are used. A hypoechoic mass continuous with the muscular propria is a key diagnostic marker, and endoscopic ultrasonography can also be useful. Nonhomogeneous lesions larger than 4 cm with irregular borders are more likely to be malignant GISTs. While biopsies may be attempted, they often have poor yields due to the submucosal location of the tumors. Horowitz et al. and Conlon et al. found that only 50% of preoperative biopsies were diagnostic [11]. Although percutaneous fine-needle aspiration/biopsy is an option, it should be reserved for cases where the diagnosis is uncertain.

Surgical resection remains the primary treatment approach, with the aim of removing the tumor completely, including any involved adjacent organs. Despite thorough surgical excision, GISTs frequently recur. Ng et al. reported that only 10% of patients were disease-free after long-term follow-up, and DeMatteo et al. found a 40% recurrence rate within two years. Hepatic involvement was observed in about two-thirds of recurrences, with the liver being the sole site in half of those cases. Local or peritoneal recurrences are also possible. Most recurrences occur within the first two years, although low-grade tumors may recur up to 10 years later. Management of recurrent disease can be challenging, with repeat curative resection potentially offering a limited survival advantage over palliative chemotherapy [11]. Evaluating risk factors is essential for both therapeutic and prognostic purposes, with primary parameters including tumor size, mitotic index, and anatomic location as outlined by the Armed Forces Institute of Pathology [12].

## Conclusions

Detecting a huge intra-abdominal retroperitoneal mass is a significant finding that warrants further investigation. The mass’s size suggests it could impact surrounding structures and potentially lead to complications. Given the diverse range of conditions that can present as a retroperitoneal mass, additional diagnostic evaluations are essential. Imaging techniques such as CT scans, MRIs, and USs can provide valuable insights into the mass's location, size, and characteristics. However, a definitive diagnosis often requires a tissue biopsy or surgical exploration. Managing a huge intra-abdominal retroperitoneal mass typically involves a multidisciplinary approach. As demonstrated in our case, consultations with surgical specialists, oncologists, and other relevant experts may be necessary to develop a tailored treatment plan. In summary, a substantial intra-abdominal retroperitoneal mass requires comprehensive evaluation and management. Prompt consultation with healthcare professionals is crucial for accurately diagnosing the masses and implementing effective treatment strategies to achieve the best possible outcomes.
